# Freiburg Neuropathology Case Conference: A 54-Years-Old Patient with Fatigue, Headaches, and Personality Changes

**DOI:** 10.1007/s00062-024-01419-x

**Published:** 2024-05-16

**Authors:** M. Frosch, A. J. Braun, J. Grauvogel, M. Prinz, H. Urbach, D. Erny, C. A. Taschner

**Affiliations:** 1 Department of Neuropathology, Medical Centre—University of Freiburg, Breisacherstraße 64, 79106 Freiburg, Germany; 2 Department of Neuroradiology, Medical Centre—University of Freiburg, Freiburg, Germany; 3 Department of Neurosurgery, Medical Centre—University of Freiburg, Freiburg, Germany; 4 Faculty of Medicine, University of Freiburg, Freiburg, Germany

**Keywords:** Meningeoma, Primary central nervous lymphoma, Hemangiopericytoma/Solitary fibrous tumor, Dural-based metastasis, Dural cavernous hemangioma, Dural-based glioblastoma, Neurosarcoidosis

## Case Report

A 54-year-old male patient presented with symptoms of fatigue, headaches and changes in personality. The neurological status on admission to hospital was unremarkable.

Computed tomography (CT) and magnetic resonance imaging (MRI) revealed a large space-occupying bifrontal tumour. Due to the clinical symptoms and the space-occupying effect of the lesion, surgery was indicated.

The operation was performed under general anaesthesia with the patient in supine position and the head fixed in the Mayfield clamp. A bifrontal craniotomy was performed, revealing a partly exophytic tumour with destruction of the frontal calvarium. The dura was incised and the infiltrated superior sagittal sinus was ligated and cut. The reddish-beige tumour with a solid consistency could then be dissected from the frontal cortex and completely removed. The tumour-infiltrated dura was resected and a duraplasty with DuraGen patch and fibrin glue was performed. The infiltrated bone was also removed and a polymethyl methacrylate (PMMA) cranioplasty was inserted.

Postoperatively, the patient showed no new neurological deficits, the further postoperative course was unremarkable and the patient was discharged home on the 8th postoperative day.

## Imaging

**Fig. 1:**
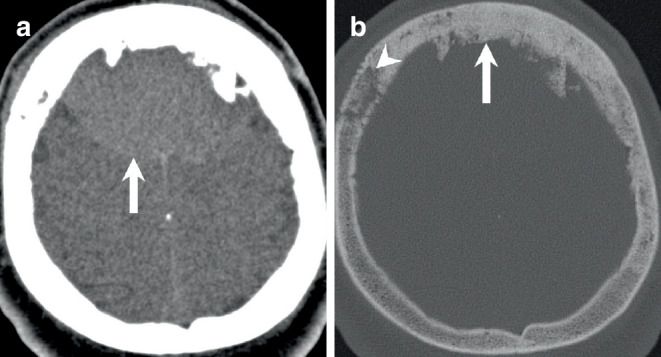
Axial CT images in soft tissue settings (**a**) obtained upon admission revealed a bifrontal space-occupying lesion, demonstrating slightly higher density compared to brain tissue (arrow). On axial CT images in bone window settings (**b**), the frontal bone appeared denser and hyperplastic (arrow) with evident osteolytic areas (arrowhead)

**Fig. 2:**
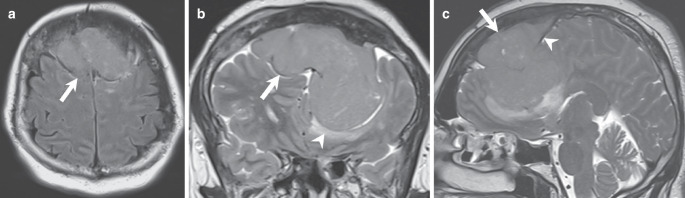
Axial (**a**) fluid attenuated inversion recovery (FLAIR) images, as well as coronal (**b**), and sagittal (**c**) T2 weighted images confirmed the presence of an extra-axially located bifrontal lesion displaying homogeneous isointensity relative to the cortex (**a–c**, arrow). Perifocal edema was observed along the margins of the basal aspects of the lesion (**b**, arrowhead). Portions of the lesion (**c**, arrow) were clearly situated underneath the dura mater (**c**, arrowhead)

**Fig. 3:**
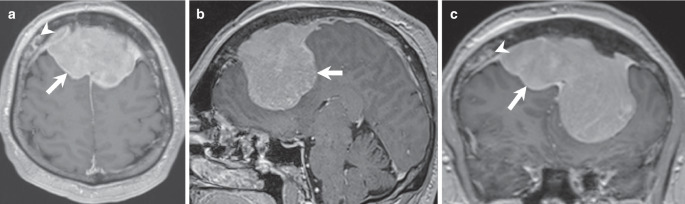
Axial (**a**), coronal (**b**), and sagittal (**c**) T1-weighted images, acquired post-gadolinium (Gd) administration, showed uniform contrast enhancement of the lesion (**a–c**, arrow). The osteolytic areas seen on the CT scan also showed homogeneous enhancement of contrast (**a,** **c**, arrowhead)

**Fig. 4:**
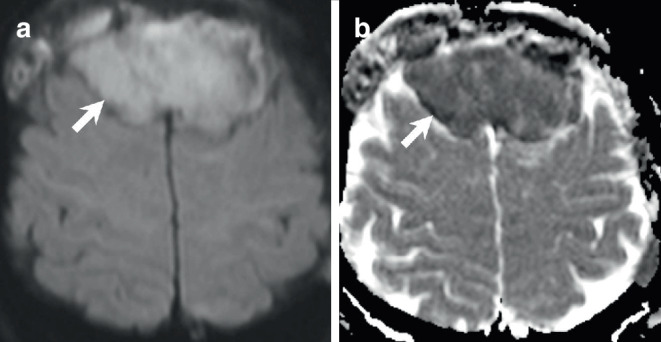
Axial diffusion weighted images with b 1000 maps (**a**) and apparent diffusion coefficient maps (**b**) displayed generalized diffusion restriction within the lesion (**a,** **b**, arrow), indicative of a hypercellular tumour

The initial cranial computed tomography (CT) scan upon admission revealed a bifrontal space-occupying lesion, demonstrating slightly higher density compared to brain tissue on soft tissue images (Fig. 1a, arrow). On bone window images, the frontal bone appeared denser and hyperplastic (Fig. 1b, arrow) with evident osteolytic areas (Fig. 1b, arrowhead). The lesion was distinctly located extra-axially on T2-weighted images, displaying homogeneous isointensity relative to the cortex (Fig. 2, arrow). Perifocal edema was observed along the margins of the basal aspects of the lesion (Fig. 2b, arrowhead). Portions of the lesion (Fig. 2c, arrow) were clearly situated beneath the dura mater (Fig. 2c, arrowhead). Furthermore, on T1-weighted images following gadolinium (Gd) administration, the lesion exhibited uniform contrast enhancement (Fig. 3, arrow). Note the contrast enhancement within the frontal bone corresponding to the osteolytic areas seen on the CT scan (Fig. 3a, c, arrowhead). Additionally, diffusion-weighted MR images (Fig. 4, arrow) indicated generalized diffusion restriction within the tumour.

## Differential Diagnosis

### Meningioma

Meningiomas are dural-based masses of varying size and, depending on subtype and WHO grade, differing signal intensities (SI) and contrast enhancement. While meningothelial meningiomas mainly present as T1 weighted (T1w) and T2 weighted (T2w) isointense masses, angiomatous meningiomas often show high T2w SI and strong homogenous contrast enhancement [1]. Histopathologically mixed meningeomas with fibroblastic and psammomatous components often show mixed SI as well as mild or moderate contrast enhancement [1]. Especially in higher grade meningiomas MRI features such as necrosis, haemorrhage or strong surrounding oedema are more common and should raise concerns for a higher grade mass and therefore potentially differing therapeutical procedures [2]. In order to prevent misdiagnosis as meningioma, it is very important to have awareness of mimic lesions such as lymphoma, schwannoma and hemangiopericytoma that are known to mimic meningiomas especially in the setting of a convexity or parafalcine masses [3]. While there are multiple features to possibly differentiate a meningioma from a meningioma mimic, the absence of a dural tail seems to be the best indicator for a meningioma mimic [3].

In our case, we hold meningioma to be a valid differential diagnosis in the setting of an extraaxial bifrontal mass with strong and rather homogenous contrast enhancement and mild peritumoral edema, while the moderate T2w-hyperintensity and hypoperfusion should eventually raise concern for a meningioma mimic.

### Primary Central Nervous Lymphoma

Primary central nervous lymphoma (PCNSL) occur mostly in a deep intraparenchymal location while primary extra-axial manifestation is uncommon [4]. In general, PCNSL have a predilection for the periventricular regions, mostly abutting the surface of the ventricles and/or the meninges [5]. Most CNS lymphoma show characteristic features to some extent, namely diffusion restriction, relative hypoperfusion as well as a decrease of N‑acetyl aspartate (NAA), the presence of lactate and lipid peaks and increased choline/creatinine in MR spectography [5].

In our case, we view PCNSL as a valid differential diagnosis due to the strong and homogenous contrast enhancement as well as the relative hypoperfusion of the mass. However, the extra-axial location of the mass and the rarity of primary meningeal PCNSL as an entity kept us from choosing lymphoma our top differential diagnosis above meningioma.

### Hemangiopericytoma/Solitary Fibrous Tumor

Hemangiopericytoma or Solitary fibrous tumors (SFTs) are rare, non-meningothelial mesenchymal tumors that are thought to arise from spindle cells in the vicinity of blood vessels. They rarely present as intracranial tumors, accounting for less than 1% of all intracranial tumors [6]. Mostly presenting as dural-based masses, SFTs were formerly thought to be a subtype of meningioma, but are now considered an own entity even though imaging features often are indistinguishable. SFTs typically present as T1w iso-intense with gray matter and and iso-intense to mildly hyperintense T2w lesions with often heterogeneous contrast enhancement and dural attachment [6]. In neuroimaging, bone erosion and peripheral feeding vessels are more likely to occur in SFTs than in meningiomas and can be a useful diagnostic clue in differentiating the two entities [7].

In our case, we hold SFT to be a valid differential diagnosis in the setting of a dural-based bifrontal mass with slight T2W hyperintensity and partial erosion of the adjacent frontal bone, even though the contrast enhancement in SFTs is usually heterogeneous and hyperostosis of the adjacent bone is rather untypical.

### Dural-Based Metastasis

Dural-based metastases are a relatively common entity that can mimic a meningioma and are being found in about 8–9% of patients with advanced systemic cancer, even though the exact incidence is remains unknown since it most commonly occurs in the setting of leptomeningeal involvement [8]. In neuroimaging, dural-based metastases are often indistinguishable from meningiomas even though some studies have shown that pulsed arterial spin labeling (pASL) can help differentiate the entities by showing higher intratumoral vascularity in meningiomas [9].

In the absence of any known primary malignancy in our patient, we view a dural-based metastasis as a valid but not as the most likely differential diagnosis.

### Dural Cavernous Hemangioma

Cavernous hemangioma are rare benign vascular tumors that are far more likely to be found within in the brain parenchyma, while only some cases of dural-based presentations exist in the literature [10]. Differing from typical parenchymal cavernous hemangiomas, dural-based hemangiomas less likely show a hemosiderin-rich surrounding but are more prone to mimick meningiomas or SFTs by showing a dural tail sign on contrast enhanced imaging [10]. While meningiomas typically shows hyperostosis in the adjacent osseous structures like the calvarium, cavernous hemangiomas seem to be more likely to cause focal erosions which may be a helpful clue in differentiating the entities [10].

Due to its rarity, we view dural cavernous hemangioma as a valid, but not very likely differential diagnosis.

### Dural-Based Glioma/Glioblastoma

While high-grade Gliomas/Glioblastomas can sometimes extend into the subarachnoid spaces or the leptomeninges, they very rarely show firm attachment to the dura [11]. They very rarely show a dural tail sign on contrast enhanced imaging and often present as heterogeneous masses with a pronounced peritumoral edema, why in our case, we view high-grade glioma/Glioblastoma as a rather unlikely differential diagnosis.

### Neurosarcoidosis

Neurosarcoidosis most often occurs in the setting of systemic disease, while isolated Neurosarcoidosis is seen in only 10–20% of patients [12]. While the most common finding in Neurosarcoidosis is leptomeningeal disease with diffusely thickened and nodular-enhancing leptomeninges in the basal cisterns on contrast-enhanced imaging, it may also present as parenchymal, infundibular or pachymeningeal/dural disease [12]. In dural-based presentation of Neurosarcoidosis, it most often shows single or scattered multifocal nodules and rather rarely exhibits smooth and linear margins [12].

In the absence of known Sarcoidosis in the patients clinical history and regarding the smooth, linear margins of the mass as well as the hyperostosis off the adjacent frontal bone, which is considered atypical in the setting of Neurosarcoidosis [12], we hold dural-based Neurosarcoidosis as a possible but unlikely differential diagnosis.

## Histology and Immunohistochemistry

**Fig. 5:**
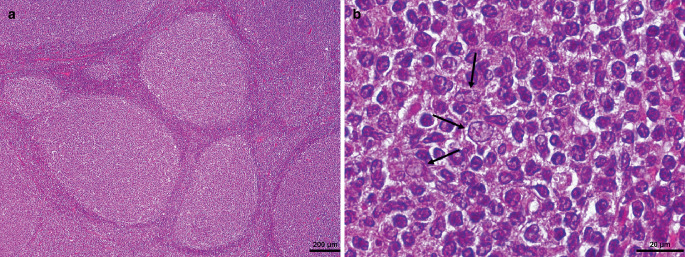
Hematoxylin-eosin (H&E) stained section (**a**) that shows a high cellular tumor that grows in a follicular pattern. Among the neoplastic cells (**b**) are predominantly centrocytes (smaller, slightly elongated, medium-dense chromatin) and single centroblasts (large, round vesicular neucleus, multiple nucleoli; arrows). Scale bar: 20 µm

**Fig. 6:**
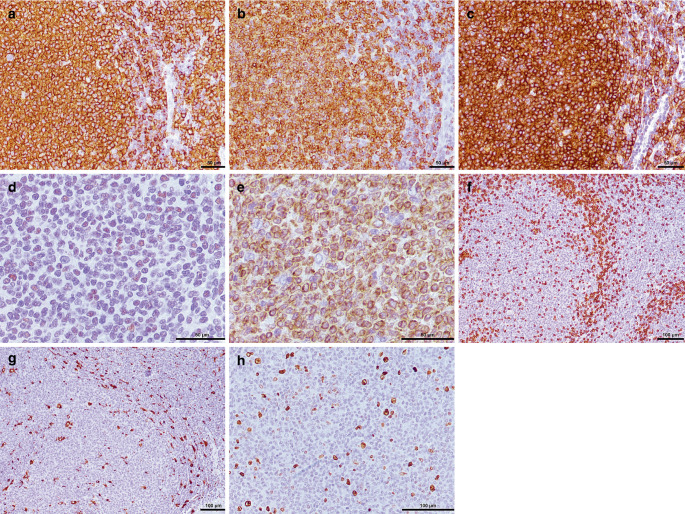
Immunohistochemistry for CD20 (**a**) showed immunoreactivity in all tumor cells. Scale bar: 50 µm. Moreover, most tumor cells express CD79a (**b**). Scale bar: 50 µm. The tumor cells exhibit strong expression of CD10 (**c**, indicating germinal center B cell origin), BCL6 (**d**, weak), and BCL2 (**e**). Scale bars: 50 µm. The tumor microenvironment comprises various other immune cells, including T cells (**f**, CD3) and macrophages (**g**, CD68). Scale bars: 100 µm. Staining against the proliferation marker Ki-67 (**h**) shows around 10% positive tumor cells. Scale bar: 50 µm

In the hematoxylin-eosin (H&E) stained sections of the formaldehyde-fixed and paraffin-embedded biopsy material, fragments of a highly cellular lymphoid tumor with a strong follicular growth pattern were found (Fig. 5a). Focally, collagen-rich connective tissue appears, corresponding to the Dura Mater. Higher magnification of the follicular structures revealed a mixture of predominantly centrocytes and single centroblasts. The former appear smaller and slightly elongated with medium-dense chromatin (less dense than mature lymphocytes but more dense than centroblasts) and a pale, hardly definable cytoplasm. In contrast, centroblasts are large cells with a round vesicular nucleus with often multiple nucleoli and a pretty visible cytoplasm (Fig. 5b). Hence, the tumor cells within the follicle structures mirrored the cellular composition of germinal centres. The tumor cells were mature B cells, labelled positive in the immunohistochemical reactions for CD20 and CD79a (Fig. 6a, b). Moreover, they showed expression of germinal centre associated markers CD10 (Fig. 6c) and BCL6 (Fig. 6d). In addition, most of the tumor cells expressed BCL2 (Fig. 6e). CMYC overexpression or MUM1 expression was, however, not present (not shown). In contrast, T cell markers (CD3) or plasma cell markers (CD38) remained negative within the tumor cells. The surrounding microenvironment of the follicular structures comprised various other immune cells, including T cells, B cells, and macrophages (Fig. 6f, g). Mitotic activity was moderate; only single mitotic figures appeared within the tumor. In line with moderate proliferative activity, approximately 10% of the tumor cells were marked in the immunohistochemical staining against Ki-67 (Fig. 6h). In summary, the histopathological finding of a follicularly structured, moderately proliferative tumor composed of centrocytes and centroblasts (both CD20+CD79+CD10+BCL6+) lead to the diagnosis.

## Diagnosis

### Follicular lymphoma (FL) within the dura mater

In general, FLs are neoplasms of germinal-center (GC) B cells with varying proportions of centrocytes and centroblasts. Typically, they occur within the lymph nodes and, less commonly, in the gastrointestinal tract, soft tissue, thoracic vertebrae, breast, ocular adnexa, and testes [13–15]. Involvement of the spleen and bone marrow is a common feature. While FL is a frequent lymphoma, accounting for 10–20% of all lymphomas, FLs within the CNS and its border regions are exceptionally rare [16]. Pubmed literature search yields only 15 case reports of FLs within the dura mater [17, 18].

Given the tumor localization within the meninges, the most relevant differential diagnoses comprise meningioma, metastasis, or a mucosa-associated lymphoid tissue (MALT) lymphoma of the dura (and other primary dural lymphomas). Macroscopically, FLs are firm and granular or nodular, thus mimicking the macroscopic features of a meningioma infiltrating the dura mater [19]. Meningiomas are the most common brain tumor in adults (37.6% of all CNS tumors), occurring most likely in the elderly [20]. Among the histological meningioma subtypes, the lymphoplasmacyte-rich meningioma exhibits intense lymphocyte infiltration. However, these lymphoplasmacytic infiltrates constitute an extensive chronic inflammatory component and do not represent tumor cells themselves. Meningioma tumor cells often grow in a lobular pattern and exhibit a positive signal in the immunohistochemical reaction for EMA. Together, histomorphological features (lobular versus follicular growth pattern) and immunohistochemical expression profiles (i.e., EMA versus CD20) allow a clear distinction between meningioma and FL. The same is true for (lepto-)meningeal metastases, which occur in 4–15% of patients with solid tumors [21, 22]. Here, melanoma (23%) and lung cancer (9–25%), followed by breast carcinoma, have the highest incidence rates [19]. These entities can clearly distinguished from lymphomas through their immunohistochemical marker profile (incl. cytokeratines for metastasis and melan A for melanoma).

As stated above, lymphomas arising primarily from the meninges are exceedingly rare. As the meninges are anatomically divided into leptomeninges (Pia Mater and Arachnoidea Mater) and Dura Mater, primary meningeal lymphomas can arise in both compartments. Primary leptomeningeal lymphomas are typically high-grade B cell lymphomas and only single cases of T cell lymphomas have been reported [17, 23]. More often, leptomeninges are secondarily infiltrated by lymphoma cancer cells. Leptomeningeal involvement in peripheral Non-Hodgkin’s lymphomas (NHLs) occurs in 6–8% of patients [24, 25]. The frequency of leptomeningeal infiltrates increases with the clinical aggressiveness of the tumor [26]. On the other hand, primary lymphomas of the dura are prevalently low-grade B cell lymphomas. Among them, the most common entity is the MALT lymphoma of the dura. These are composed of small lymphocytes and marginal zone cells. Since MALT lymphoma cells can replace the germinal centers, the tumor can mimic follicular lymphomas. However, the absence of a pronounced extrafollicular B cell fraction and the expression of follicular-center markers (especially CD10) supports the diagnosis of follicular lymphoma [26, 27]. Due to the small number of FL of the dura reported in the literature, prognostic evidence is limited.
